# Biomarkers for Primary Sjögren’s Syndrome

**DOI:** 10.1016/j.gpb.2015.06.002

**Published:** 2015-09-08

**Authors:** Weiqian Chen, Heng Cao, Jin Lin, Nancy Olsen, Song Guo Zheng

**Affiliations:** 1 Division of Rheumatology, The First Affiliated Hospital, College of Medicine, Zhejiang University, Hangzhou 310003, China; 2 Division of Rheumatology, Penn State University Hershey College of Medicine, Hershey, PA 17033, USA

**Keywords:** Biomarker, Fms-like tyrosine kinase 3 ligand, Myxovirus-resistance protein A, Non-coding RNAs, Saliva, Sjögren syndrome

## Abstract

Primary Sjögren’s syndrome (pSS) is a systemic autoimmune disease with exocrine gland dysfunction and multi-organ involvement. Recent progress in understanding the pathogenesis of pSS offers an opportunity to find new **biomarkers** for the diagnosis and assessment of disease activity. Screening noninvasive **biomarkers** from the **saliva** and tears has significant potential. The need for specific and sensitive **biomarker** candidates in pSS is significant. This review aims to summarize recent advances in the identification of **biomarkers** of **Sjögren syndrome**, trying to identify reliable, sensitive, and specific **biomarkers** that can be used to guide treatment decisions.

## Introduction

Primary Sjögren’s syndrome (pSS) is a chronic systemic inflammatory autoimmune disease characterized by keratoconjunctivitis sicca and xerostomia [1]. It may involve the exocrine glands of the skin, respiratory, urogenital, and digest tract [2]. Besides, extra-glandular involvement is common, including synovitis, interstitial lung disease, neuropathy, renal disease, vasculitis, and auto-immune cytopenias [2]. Activated B lymphocytes is a hallmark of the disease, which is characterized by the presence of rheumatoid factor, hypergammaglobulinemia, and autoantibody to Ro/Sjögren’s-syndrome-related antigen A (SSA) and La/Sjögren’s-syndrome-related antigen B (SSB) [3]. Additionally, there is a 6.5-fold increased risk of non-Hodgkin’s lymphoma in pSS, which is much higher than other autoimmune diseases [4]. Primary SS occurs alone with more restricted symptoms such as sicca, while secondary SS (eSS) was related to other autoimmune diseases, having more symptoms of original disease other than exocrine glands’ dysfunction [2,5].

The non-specific symptoms such as sicca, fatigue, and arthralgia usually are ignored by patients, leading to delayed diagnosis, to a mean of 7 years [6]. At diagnosis, pSS patients are characterized by high titer of antinuclear antibodies and IgG antibodies that are not specific for this disease but are shared with other autoimmune diseases such as systemic lupus erythematosus (SLE) [7]. The labial salivary gland biopsy is often helpful to diagnose the pSS disease according to revised 2002 American-European criteria [5] and 2012 American College of Rheumatology (ACR) criteria [8]. However, the biopsy is an invasive procedure that is not accepted by all patients, and it may delay definitive diagnosis. For these reasons, noninvasive biomarkers for assessment and diagnosis of pSS are urgently needed. An ideal biomarker should be noninvasive, specific to diagnose, sensitive to treatment, or useful to predict disease development.

## Saliva and tears as ideal biomarker resources

Saliva may represent a crucial source containing valuable biomarkers for local and systemic disease [9]. Saliva meets the demands of a simple, inexpensive, and non-invasive sampling method, which can be repeated frequently without risks and discomfort for the individual [10]. The discovery of saliva-based molecular and immunologic biomarkers enables the use of saliva to evaluate the presence of disease. Recently, quantitative proteomics such as two-dimensional gel electrophoresis (2D-GE) or mass spectrometry (MS) technology have been utilized to identify saliva biomarkers, *e.g.*, haptoglobin hp2, zinc α2-glycoprotein, and human calprotectin in lung cancer [11].

Previous studies have already suggested saliva proteomic and genomic biomarker candidates for pSS [12], however, a sensitive and specific biomarker from this fluid source has not yet been established [13]. The protein profile of saliva is dominated by a series of highly-abundant proteins, such as salivary amylase, albumin, and immunoglobulin, which mask potential low-abundance biomarkers [14]. High-abundance proteins ideally should be depleted before application of proteomics. Two novel representative biomarkers, profiling and carbonic anhydrase I (CA-I), have been recently identified after depletion of high-abundance proteins followed by 2D-GE, quantitative dimethylation liquid chromatography tandem MS (LC-MS/MS), and Western blot. Profilin showed an average increase of 3.19-fold and CA-I a decrease of 1.5-fold in patients with pSS, compared to healthy controls [14]. Profilin was a cytoskeleton actin related protein, involved in the organization of microfilaments and required in early embryo development [15], and CA-I was an enzyme involved in tissue hydration [16]. However, it is still unclear whether such protein abnormalities have contributed to the pathogenesis of pSS. Moreover, the sensitivity and specificity of both candidates for diagnosing and evaluating the disease activity were not shown [14]. This study was also limited by the small sample size (18 pSS *vs.* 18 healthy controls for discovery, and 10 pSS *vs.* 10 healthy controls for validation) (Table 1).

A study by Delaleu et al. [17] aimed to explore biomarker signatures of pSS using a 187-plex beads and antibody-based capture assay. There was profound increment of interleukin 4 (IL-4), IL-5, and clusterin (*P* < 0.001) in the salivary proteome of 48 patients with pSS, compared to controls without pSS including 12 patients with rheumatoid arthritis (RA) and 12 healthy individuals [17]. IL-4 and IL-5 are cytokines produced by T helper 2 (Th2) cells, which are associated with T lymphocyte differentiation and B lymphocyte activation [18,19], whereas clusterin is implicated in multiple biological processes including inflammation [20]. IL-4, IL-5, and clusterin in combination, were suggested as accurate predictors 93.8% of disease [17] (Table 1). However, quantitative analysis is absent, further testing and validation of these biomarker signatures are needed.

Tear is another fluid source with potential to yield discriminatory biomarkers for assessment of systemic disease and has been proposed as a good source of biomarkers for the diagnosis of SS [21]. Cathepsin S was a cysteine endopeptidase, which may be involved in antigen presentation and immunity [22]. Recently, tear cathepsin S activity was found significantly increased (*P* < 0.0001) in patients with SS, compared to patients with other autoimmune diseases (*e.g.*, RA and SLE), patients with nonspecific dry eye disease, and healthy controls [22]. This study suggests that tear cathepsin S activity may be a simple and noninvasive biomarker for the diagnosis and evaluation of SS. However, there were no differences detected in tear cathepsin S levels between patients with pSS and those with eSS. Furthermore, tear cathepsin S activity did not correlate with the serum levels of anti-Ro/SSA or anti-La/SSB antibodies. It also failed to distinguish pSS from eSS as pointed out above [22]. More important, the authors did not give a cutoff value to diagnose the disease.

## Interferon type I signature as a valuable pSS biomarker

Accumulating evidence supports the notion that interferon (IFN) type I plays an important role in the pathogenesis of pSS [23–25]. The expression of IFN type I-inducible genes (IFN type I signature) was increased in the peripheral blood and salivary glands of patients with pSS [26]. The IFN type I signature was present in 55% of patients with pSS and overexpression of IFN type I signature is associated with higher levels of disease activity and anti-Ro/SSA or anti-La/SSB autoantibodies [27,28]. Thus, IFN type I is a potential biomarker for diagnosis and evaluation of disease activity in pSS [26].

Furthermore, Maria et al. [23] showed that myxovirus-resistance protein A (MxA) might serve as a biomarker for IFN type I activity in patients with pSS. The IFN scores, representative of total IFN type I activity, were assessed by the expression levels of certain IFN type I signature genes such as *IFI44*, *IFI44L*, *IFIT3*, *LY6E*, and *MX1* in CD14^+^ monocytes by real-time quantitative PCR. They found that IFN scores significantly correlated with protein levels of MxA in the monocytes (*r* = 0.741, *P* < 0.001) and in the whole blood (*r* = 0.764, *P* < 0.001). Interestingly, 100 μg/L was the cutoff value of MxA by enzyme immunoassay, MxA < 100 μg/L was suggested as low and MxA > 100 μg /L as high. They found that MxA levels correlated with the European League Against Rheumatism (EULAR) SS disease activity index (ESSDAI) scores and clinical laboratory profiles such as the levels of immunoglobulin (IgG, IgA, and IgM) and autoantibody (anti-Ro/SSA, anti-La/SSB, and rheumatoid factor). Finally, MxA levels were reduced in patients treated with hydroxychloroquine, which is reported to reduce IFN type I activity. Therefore, MxA level might be useful in identifying patients who respond to hydroxychloroquine therapy [23]. Nonetheless, this study was also limited by the small sample size (Table 1). Further study recruiting more patients is needed to strengthen this finding.

## Flt-3L as a potential novel biomarker for lymphoma development in pSS

Patients with pSS are at a greater risk of developing lymphoma [4]. A recent multicenter study has found clues of lymphoma that include low complement component 4 (C4), cryoglobulins, anti-La antibodies, and leukopenia. Salivary swelling and the absence of the above biomarkers provided a negative predictive value for lymphoma of 98% in patients with pSS [29].

Furthermore, Tobon et al. [30] showed that Fms-like tyrosine kinase 3 ligand (Flt-3L) might be associated with lymphoma in pSS. There were higher levels of Flt-3L in pSS patients who had a history of lymphoma. More importantly, levels of Flt-3L were associated with previously-identified risk markers for lymphoma development, such as presence of purpura and lymphocytopenia, lower levels of C4 and IgM, higher levels of β2 -microglobulin, and a higher disease activity score. Furthermore, the Flt-3L levels were increased in the serum up to 94 months (mean 46 months) before the diagnosis of lymphoma. This study has suggested an ideal cutoff value of Flt-3L (175 pg/ml) for revealing an association with lymphoma in patients with pSS (44% for sensitivity, 97.5% for specificity, and 97% for negative predictive value). It is, therefore, believed that Flt-3L is an ideal biomarker for lymphoma development in pSS [30].

B cell abnormalities are known to be present in patients with pSS [2]. The levels of CXC ligand 13 protein (CXCL13), a B cell homeostatic chemokine, were elevated in serum (162 ± 184 pg/ml *vs*. 26.8 ± 22.8 pg/ml, *P* < 0.0001) and saliva (368 ± 631 pg/ml *vs.* 26.7 ± 36.8 pg/ml, *P *= 0.0016) in patients with pSS, compared to healthy controls using the enzyme-linked immunosorbent assay (ELISA). They suggested 72.4 pg/ml and 100.3 pg/ml as an elevated level in serum and saliva, respectively. Another report found that CXCL13 was also highly expressed in SS mouse models [31]. Neutralization of CXCL13 ameliorated disease progression in a murine model [31]. Therefore, CXCL13 seems to be a promising biomarker in pSS. However, CXCL13 was not exclusively expressed in pSS. Expression of CXCL13 was also detected in lupus and correlated with lupus disease activity measures [32].

## Exploring genomic biomarkers for pSS

MicroRNAs (miRNAs) are small non-coding and single-stranded RNAs that are able to regulate gene expression post-transcriptionally [33]. miRNAs have been proposed as excellent salivary biomarker candidates due to their easy isolation and identification through quantitative PCR [34–36]. Several novel miRNAs have been described in pSS [34,37–39]. For instance, expression of miR-146a was significantly increased in pSS patients compared with healthy controls [39]. In addition, another 2 miRNAs, miR-768-3p and miR-574, were associated with minor salivary gland inflammation in 15 patients with pSS [38].

Although some studies have been accomplished in the understanding of miRNA in SS [34,37–39], the role of long non-coding RNAs (lncRNAs) remained uncharacterized. Ice et al. found significant upregulation of the 2p25.1 lncRNA in SS patients (*P* = 3.69 × 10^−5^) compared to healthy controls [40]. Furthermore, this transcript was found highly expressed in CD4^+^ and CD8^+^ T cells, and NK cells [40].

As we know, epigenetic modifications are important to control gene expression associated with pathogenesis of autoimmune disease [35]. Thabet et al. [41] reported that global DNA methylation was reduced in salivary gland epithelial cells (SGECs) from SS patients, which was associated with a 7-fold decrease in the expression of the gene *DNMT1*, which encodes the DNA methyltransferase 1, and a 2-fold increase in the expression of the gene *Gadd45a*, which encodes the growth arrest and DNA-damage-inducible protein GADD45 alpha (GADD45α). This study suggested that SGEC dysfunction in SS may be partially linked to epigenetic modifications [41], which, however, is hard to be a biomarker for diagnosing and predicting the development of disease. Taken together, whether alterations in ncRNAs or epigenetic modifications could serve as reliable biomarkers for diagnosis and evaluation of disease activity of pSS needs further study.

## Conclusion and perspective

In summary, a number of candidate saliva biomarkers have been revealed by quantitative proteomics, enabling exploration of this simple and noninvasive tool to diagnose and evaluate the pSS. There are strengths and limitations in our included studies. Totally, different kinds of biomarkers were reported such as proteins, genes, or miRNAs. The combined studies using salivary proteomics and microRNA studies may improve the sensitivity and specificity of biomarker for disease. Unfortunately technical limitations still stand in the way and saliva biomarkers that have greater sensitivity and specificity for predicting disease are needed. There were no traditional tests and validation cohorts in most studies. Given that IFN type I is likely to be involved in the pathogenesis of pSS, the observation that whole-blood MxA level correlates with IFN score and pSS activity and is reduced after treatment, supports MxA as a useful candidate biomarker to diagnose and assess activity in patients with pSS. We could explore more novel biomarkers through the IFN signal pathway. Interestingly, Flt-3L could serve as a useful marker to predict the risk of lymphoma in patients with pSS, with a high specificity and reasonable sensitivity, as well as a high negative predictive value. Further study should concentrate on exploring the reliable biomarker from saliva using simple methods. It is likely that those novel biomarkers with utility as diagnosis and assessment tools for pSS may become a reality in the near future.

## Competing interests

The authors have declared no competing interests.

## Figures and Tables

**Table 1:** Summary of biomarker studies for patients with pSS

**Biomarker**	**Source**	**Method**	**No. of pSS patients (controls)**	**Average ratio (pSS/control)**	***P* value**	**Sensitivity (%)**	**Specificity (%)**	**Ref.**
Profilin	Saliva	Western blot	10 (healthy 10)	3.19/1	<0.05	NA	NA	[14]
Anhydrase I		1/1.5	<0.05	NA	Also found in SLE
IL-4	Saliva	MAPs	48 (RA 12, healthy12)	NA	<0.001^§^	93.8^‡^	95.8^‡^	[17]
IL-5		<0.001^§^
Clusterin		<0.001^§^
Cathepsin S	Tear	BioVision Kit	28 (healthy 33)	37.8/1	<0.0001	95.4^#^	NA	[22]
	28 (eSS 45)	∼ 1/1	0.84	NA	NA
MxA	Whole blood	EIA	21 (healthy 27)	202.4/1	<0.001	NA	NA	[23]
Flt-3L	Serum	ELISA	18/369/50^※^	2.34/1.54/1	<0.0001	44.4^†^	97.5^†^	[30]
CXCL13	Serum	ELISA	27 (healthy 21)	6.04/1	<0.0001	56	NA	[31]
Saliva	ELISA	29 (healthy 20)	13.78/1	<0.01	57	NA	

*Note:* pSS, primary Sjögren’s syndrome; NA, not applicable; SLE, systemic lupus erythematosus; IL, Interleukin; MAPs, multiplexing antibody-based sandwich-immunoassays; RA, rheumatoid arthritis; §, calculated by Student’s t-test; ‡, calculated by the discriminant function analysis; eSS, secondary Sjögren’s syndrome; #, both pSS and eSS; MxA, myxovirus-resistance protein A; EIA, enzyme immunoassay; Flt-3L, Fms-like tyrosine kinase 3 ligand; ELISA, enzyme-linked immunosorbent assay; ※, 18 pSS patients with lymphoma, 369 pSS patients without lymphoma and 50 healthy controls; †, the association of lymphoma in patients with pSS was calculated by the receiver operating characteristic curve (ROC) analysis.
